# Identifying Potential Breast Carcinogens: A New Approach

**DOI:** 10.1289/ehp.123-A305

**Published:** 2015-12-01

**Authors:** Wendee Nicole

**Affiliations:** Wendee Nicole has written for *Discover*, *Scientific American*, and other publications.

In 2007 the National Academy of Sciences called for a paradigm shift in how chemicals are tested, recommending that toxicity testing look not just at disease end points such as tumors or birth defects but also at “upstream” events, meaning early changes in developmental processes that may later lead to disease.^1^ In this issue of *EHP*, a team of researchers apply that recommendation in a novel framework in which they work backward from a specific health outcome—in this case, breast cancer—through the biological mechanisms associated with it to identify appropriate assays for disease-specific chemical risk assessment.^2^

The Hazard Identification Approach for Breast Carcinogens (HIA-BC) was developed by the University of California (UC) Berkeley Breast Cancer and Chemicals Policy project. An interdisciplinary panel of 18 scientists combed the breast cancer literature and cataloged molecular, cellular, and tissue changes that are strongly associated with the disease. These biological changes fell under three broad categories: endocrine disruption, alterations to mammary gland development, and processes generally associated with cancer, including cell cycle changes and genotoxicity (ability to cause DNA damage).^3^

**Figure:**
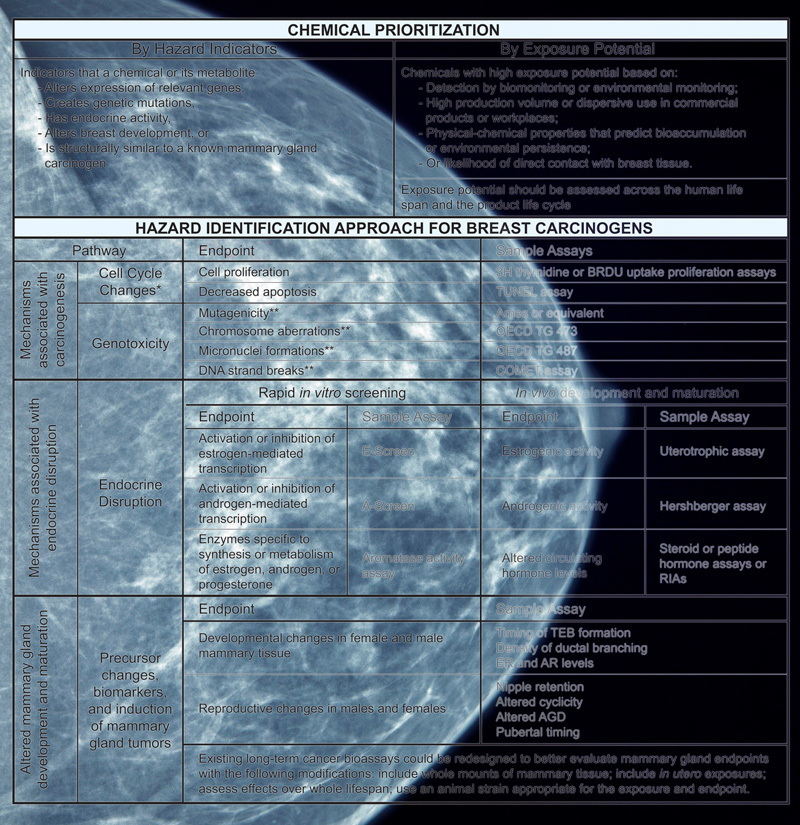
A new testing framework provides a disease-specific guide for assessing potential breast carcinogens. Mammogram: © Mark Kostich/iStockphoto; framework: Schwarzman et al. (2015)^1^

The panel then identified *in vivo* and *in vitro* assays capable of detecting each of these biological alterations and assembled these in the HIA-BC framework. Rather than requiring specific assays, the HIA-BC provides examples of relevant tests for each end point—an acknowledgment that best practices and methodologies change over time.

The authors pilot tested the HIA-BC using 11 well-studied chemicals. Most were known or suspected breast carcinogens, but the list also included one linked to cancer but not specifically to breast cancer (arsenic) and one with no evidence of human carcinogenicity (caprolactam). The use of well-studied chemicals enabled the investigators to determine whether the HIA-BC would correctly identify test compounds as potential breast carcinogens.

All the known carcinogens tested positive on multiple assays in the HIA-BC, which in real-world use would flag these chemicals for more in-depth study. The pilot test revealed numerous data gaps even for these relatively well-studied chemicals, mainly in endocrine disruption and mammary gland development effects. Almost no chemical had gone through the full battery of toxicology tests for genotoxicity, endocrine disruption, mammary gland development, and cellular modifications linked to cancer.^2^

The pilot test also identified gaps in federal high-throughput chemical screening programs, including the Environmental Protection Agency’s ToxCast™ and Endocrine Disruptor Screening Program, the High Throughput Screening Initiative of the National Toxicology Program, and the interagency Tox21 Initiative. These programs rely on *in vitro* assays that—while useful—may not be specific to some of the mechanisms most relevant to how breast cancer develops. “Although there is significant overlap,” the authors write, “the national screening programs could increase their relevance to breast cancer by adding several new end points, including *a*) Her2 activation, *b*) progesterone receptor activity, *c*) prolactin effects, *d*) comprehensive coverage of ERβ activity, and *e*) expression of additional genes that are relevant to breast cancer.”^2^

“Breast cancer is the second most common cancer in the world and by far the most frequent cancer among women. At the same time, relatively few known causes of breast cancer have been identified,” says Kathryn Guyton, senior toxicologist in the International Agency for Research on Cancer  Monographs Programme in France. The proportion of breast cancer attributed to environmental chemicals remains unknown, but since only 5–20% can be linked to inherited genes,^4^ environmental factors likely play a significant role. “This study could help to bridge that gap by proposing an intriguing and novel screening strategy for breast cancer carcinogens,” Guyton says.

There is evidence that both genotoxic and nongenotoxic mechanisms drive the development and growth of breast cancer—a consideration that is built into the HIA-BC framework. And although this testing scheme focuses on breast cancer, the authors say the same approach could be used to develop hazard assessment schemes relevant to other diseases as well.

“We really have the chance, by improving the way we’re testing chemicals, to detect those that are setting us up for future disease,” says lead author Megan Schwarzman, a physician and environmental health researcher at UC Berkeley. “I’m eager to see the work continue, and continue relative to other diseases, so it can enrich the chemical testing being done on the national level.”
